# Unusual presentations of functional parathyroid cysts: a case series and review of the literature

**DOI:** 10.1186/s13256-017-1502-1

**Published:** 2017-11-29

**Authors:** Youssef El-Housseini, Martin Hübner, Ariane Boubaker, Jan Bruegger, Maurice Matter, Olivier Bonny

**Affiliations:** 1 Service of Nephrology and Hypertension, Lausanne University Hospital, Rue du Bugnon 17, 1011 Lausanne, Switzerland; 2 Service of Visceral Surgery, Lausanne University Hospital, Lausanne, Switzerland; 3 Service of Nuclear Medicine, Lausanne University Hospital, Lausanne, Switzerland

**Keywords:** Hyperparathyroidism, Parathyroid cyst, PTH, Parathyroid hormone, Case report

## Abstract

**Background:**

Cysts of parathyroid origin are sometimes encountered and can easily be mistaken as thyroidal cysts. Functional parathyroid cysts, with symptoms and signs of hyperparathyroidism, are rare and may be a diagnostic challenge to clinicians. We report here on three cases of functional parathyroid cysts that illustrate diagnosis difficulties related to unusual clinical presentations in three Caucasian women, including negative parathyroid scintigraphy.

**Case presentations:**

**Patient 1**, an 87-year-old Caucasian woman presented with confusion and dysphagia. She had hypercalcemia and elevated parathyroid hormone levels suggesting primary hyperparathyroidism. Parathyroid scintigraphy did not reveal any focal uptake, but a computed tomography scan of her neck identified a large cyst in the upper right thyroid region. At cervicotomy, a parathyroid cystic adenoma was removed. **Patient 2**, a 31-year-old Caucasian woman was investigated after a hypertensive crisis related to primary hyperparathyroidism. Cervical ultrasound identified a large cystic lesion in the lower left thyroid lobe that was removed by minimally invasive cervicotomy. **Patient 3**, a 34-year-old Caucasian woman presented with an indolent growing mass of the neck and a past medical history of kidney stones. Primary hyperparathyroidism was diagnosed. Ultrasound showed a cystic mass, but parathyroid scintigraphy was negative. Cervical exploration revealed a large cystic adenoma, containing high parathyroid hormone levels.

**Conclusions:**

Diagnosis of functional parathyroid cysts can be challenging due to various clinical presentations and negative parathyroid scintigraphy. Surgery, but not fine-needle sclerotherapy, appears to be the safest treatment option. Despite its rarity, differential diagnosis of cystic lesion of the neck should include primary hyperparathyroidism due to functional parathyroid cysts.

## Background

Cysts of the parathyroid gland are traditionally classified as either non-functional or as functional and may represent up to 5% of all cystic tumors of the anterior neck [1]. The majority of parathyroid cysts are non-functional, generally discovered during thyroid or cervical investigations, and are not associated with primary hyperparathyroidism [2]. By contrast, functional cysts induce symptoms and signs of primary hyperparathyroidism. Their fluid contains high concentrations of parathyroid hormone (PTH), which may induce parathyroid crisis in case of rupture. Here, we report three cases of patients with functional parathyroid cysts, with atypical presentation, and we propose a simple investigation and treatment algorithm.

## Case presentation

We reviewed 10 years of activity (2002 to 2012) of parathyroid surgery at our university hospital, a tertiary referral center for endocrine surgery. The rationale for the starting point was the standardization of diagnostic work-up and surgical technique in 2002: systematic double-phase parathyroid scintigraphy with technetium 99 m (^99m^Tc)-sesta methoxyisobutylisonitrile (sestamibi) for primary hyperparathyroidism and introduction of minimally invasive focused surgery. During this time period, 187 patients underwent parathyroidectomy for primary hyperparathyroidism and 32 patients for secondary or tertiary hyperparathyroidism. Three patients with parathyroid cysts were identified during this period (1.4% of all cases of operated hyperparathyroidism).

### Case 1

**Fig. 1:**
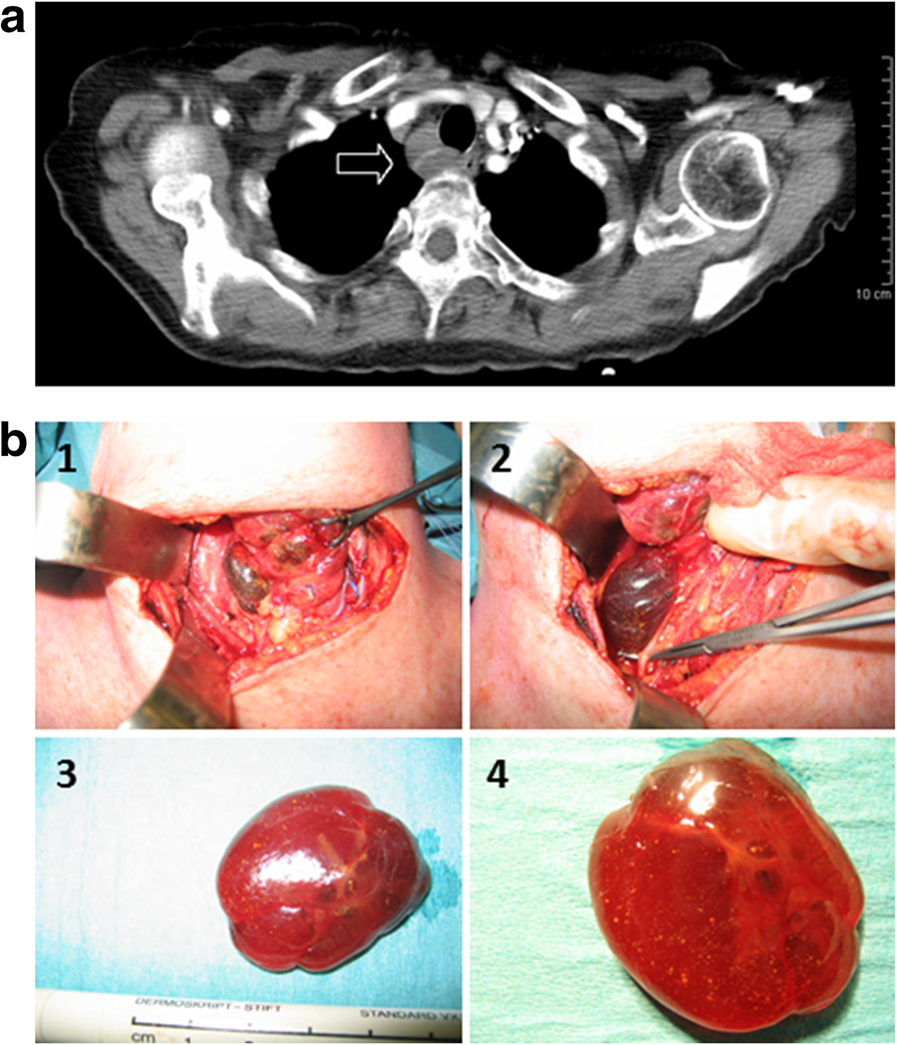
Patient 1. **a** Cervical computed tomography scan showing a paratracheal and paraesophageal cystic tumor with compression of the esophagus causing dysphagia (*arrow*). **b** Intraoperative view of the parathyroid cyst of patient 1 (panels **1** and **2**). The cyst is located to the inferior thyroid artery and the laryngeal nerve. Right thyroid lobe is retracted to the left. Panels **3** and **4** Resected cyst with fine lining of parathyroid tissue

An 87-year-old Caucasian woman presented to our emergency room with a 10-day history of progressive mental confusion and dysphagia. On admission, her blood pressure (BP) was 107/72 mmHg, pulse rate 80 beats/minute, and temperature was 36.5 °C. She was disoriented and dehydrated. Blood tests revealed hypercalcemia (13.8 mg/dl; normal range, 8.6 to 10.2 mg/dl), elevated PTH levels (305 pg/ml; normal range, 10 to 70 pg/ml), and concomitant low levels of 25-hydroxyvitamin D (25-OH vitamin D). Her renal function was impaired with estimated glomerular filtration rate (GFR) at 36 ml/minute per 1.73 m^2^. A cervical computed tomography (CT) scan (Fig. 1a and Table 1) identified a right-sided cystic nodule. Double-phase parathyroid scintigraphy with single-photon emission CT (SPECT)-CT was negative. Hypercalcemia improved with pamidronate treatment, but her plasma PTH remained high despite vitamin D supplementation. During cervicotomy, three normal-sized parathyroid glands were detected (upper and lower left and lower right), and confirmed by frozen sections. Deep behind her right inferior thyroid artery, a 3.5 × 3 × 2 cm cystic tumor filled with colloid-like fluid was carefully removed (Fig. 1b). Histopathological analysis confirmed a parathyroid adenoma with cystic transformation. She developed transient postoperative hypocalcemia, requiring calcium and 1,25-dihydroxyvitamin D3 (1,25-(OH)_2_ vitamin D3) substitution. Normalization of calcium and PTH levels was associated with full recovery, including normal mental status. At 6 months, she was fully active and had recovered from renal insufficiency.

**Table 1:** Clinical characteristics of the patients

Age, sex (years)	Clinical symptoms	Serum calcium (mg/dl)	Serum PTH (pg/ml)	Intracystic PTH (pg/ml)	Detection technique	Position of the parathyroid cyst	Size (cm)	Treatment	Serum PTH 1 day after surgery (pg/ml)
87, F	Confusion Dysphagia Dehydration	13.84	305	n.d.	MIBI negative and CT scan positive	Upper right	3.5 × 3 × 2	Surgical resection	8
31, F	Hypertensive crisis with transient ischemic attack	10.76	259	n.d.	MIBI positive and US positive	Lower left	3.5 × 2 × 1	Surgical resection	7
34, F	Kidney stones, neck mass	11.04	1410	4,347,000	MIBI suspect and US positive	Lower left	5 × 4.5 × 4*	Surgical resection after initial fine-needle aspiration with recurrence 1 month later	17

*CT* computed tomography, *MIBI* ^99m^Tc sestamibi, *n.d.* not determined, *US* ultrasound. * after preoperative fine-needle aspiration. Normal range for serum calcium is 8.6 to 10.2 mg/dl (to change units in mmol/l, divide by 4); parathyroid hormone is 10 to 70 pg/ml

### Case 2

**Fig. 2:**
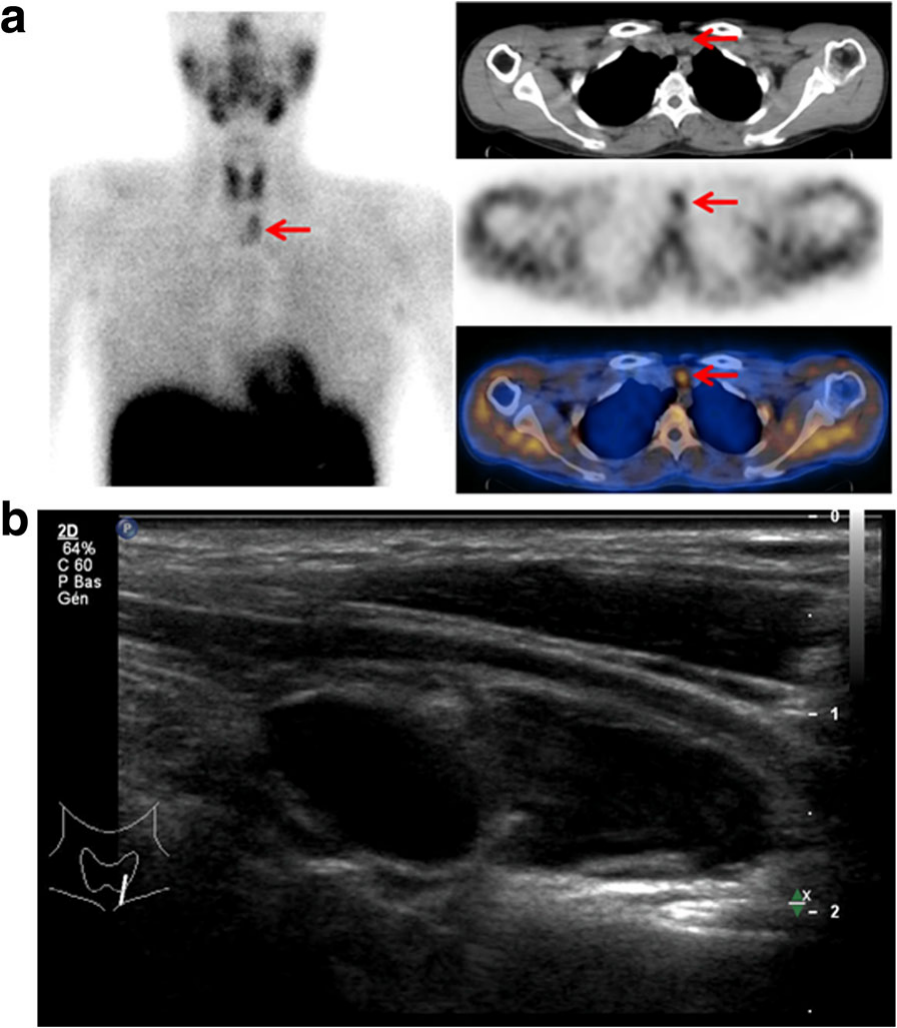
Patient 2. **a** Technetium 99 m sesta methoxyisobutylisonitrile scintigraphy (planar and single-photon emission computed tomography-computed tomography) showing a focal tracer retention localized under the left lower thyroid lobe just above the manubrium and anterior to the trachea (*red arrows*). **b** Cervical ultrasound with longitudinal view of a mixed solid and cystic nodule

A previously healthy 31-year-old Caucasian woman was investigated after acute transient ischemic attack attributed to a hypertensive crisis. Investigations excluded renovascular or adrenal causes for her hypertension. However, hypercalcemia and raised PTH levels (169 pg/ml; normal value, 10 to 70 pg/ml) suggested a diagnosis of hyperparathyroidism. A cervical ultrasound (US) showed an isolated 2 cm mixed solid and cystic nodule. Double-phase parathyroid scintigraphy with SPECT-CT revealed a lower left focal uptake and ^99m^Tc-sestamibi retention consistent with an adenoma (Fig. 2a). A left lower parathyroidectomy was successfully performed through a minimally invasive focused cervicotomy and intraoperative PTH levels went back to normal values within 15 minutes after cyst removal. At 2-week follow-up, her calcium levels and BP had returned to normal ranges. Histopathological analysis showed parathyroid adenoma with cystic transformation, surrounded by thymic tissue composed of pseudocysts lined with parathyroid cells. Six months later, she had fully recovered.

### Case 3

**Fig. 3:**
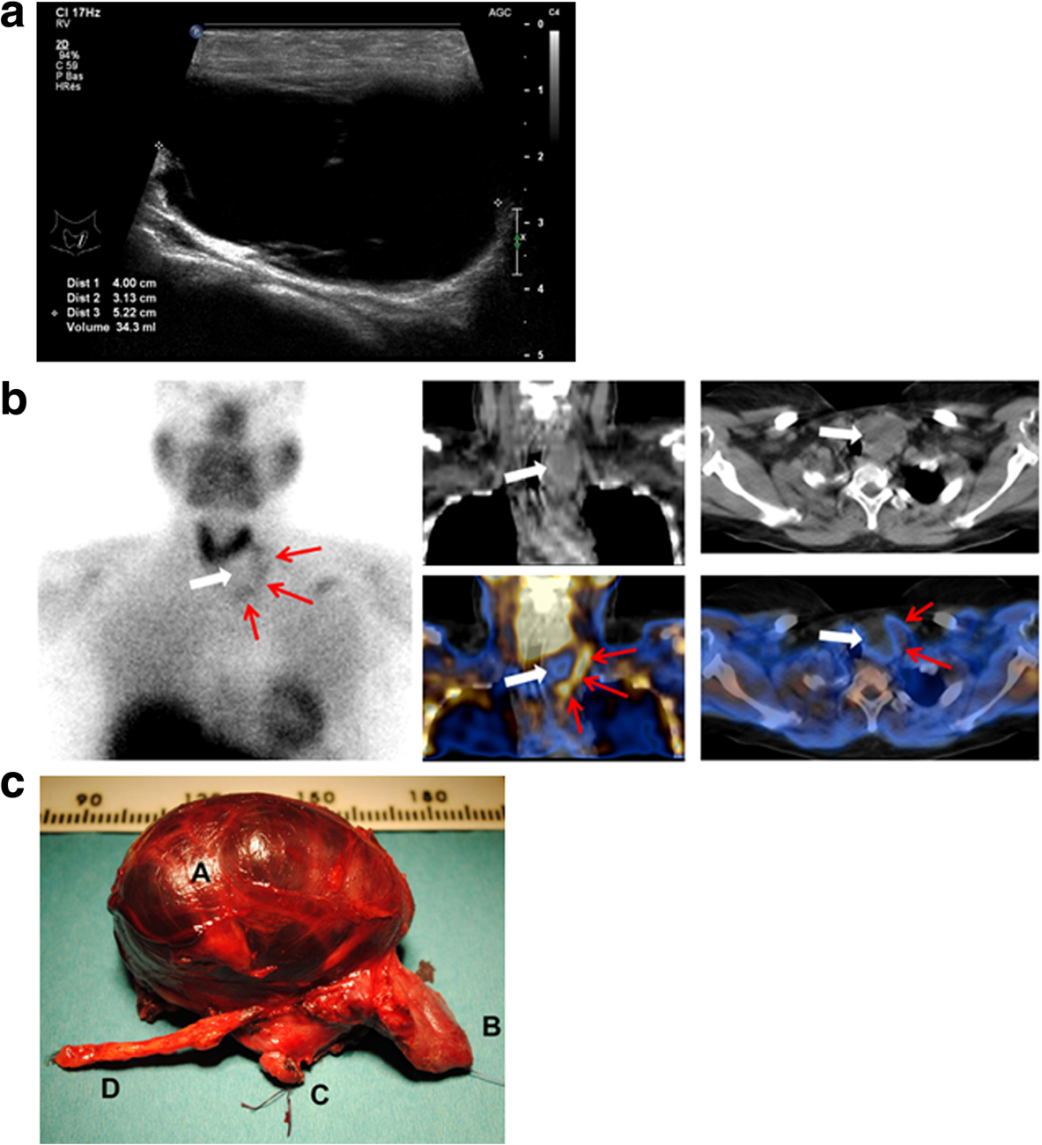
Patient 3. **a** Cervical ultrasound and longitudinal view of a large cyst. **b** Technetium 99 m sesta methoxyisobutylisonitrile scintigraphy with single-photon emission computed tomography-computed tomography. A central “cold” area (*white arrow*) corresponding to the parathyroid cyst is surrounded by faint tracer retention (*red arrows*) corresponding to the displaced parathyroid parenchyma. **c** Left thyroid lobe *en bloc* with the parathyroid cyst. *A* Cyst, *B* upper part of otherwise normal looking thyroid gland, *C* isthmus, *D* thymic remnant

A 34-year-old Caucasian woman who was obese, smoked tobacco, hypertensive, and diabetic underwent ambulatory work-up for bilateral kidney stones. She was treated by candesartan 8 mg daily, gliclazide 30 mg daily, and combined metformin 1000 mg/sitagliptin 50 mg twice a day. She also complained of a rapidly growing mass in the left side of her neck. A physical examination showed obesity with a body mass index (BMI) of 32 kg/m^2^ and revealed a soft and mobile mass on the left side of her neck. Her BP was 132/88 mmHg and heart rate was 98 beats/minute. Her initial PTH level was 557 pg/ml (normal value 10 to 70 pg/ml) and plasma calcium was 11 mg/dl. Despite correction of low 25-OH vitamin D levels, her PTH had increased to 1410 pg/ml 2 months later. A cervical US identified a nodular and multicystic tumor embedded in her left thyroid lobe (Fig. 3a). Double-phase parathyroid scintigraphy with SPECT-CT and thyroid scintigraphy showed homogenous thyroid uptake, but identified a hollow zone with a rim of tracer uptake under her left thyroid lobe (Fig. 3b), suggesting the presence of a large parathyroid cyst. US-guided puncture of the cyst revealed a PTH concentration of 4,347,000 pg/ml in the fluid, and normal thyroid hormone levels. During cervicotomy, a large left thyroid lobe with mediastinal extension was discovered and an *en bloc* left thyroidectomy (Fig. 3c) was performed, with uneventful recovery. Histopathological examination showed a 5 × 4.5 × 4 cm large cyst lined by parathyroid cells and normal thyroid gland. At 6-month follow-up, she had normal PTH and plasma calcium levels.

## Discussion

These three cases of functional parathyroid cysts are unusual in several aspects: (i) functional parathyroid cysts are rare, especially in women, representing less than 1% of primary hyperparathyroidism; (ii) cysts localized in the superior parathyroid glands (case 1) are less frequent than lower localizations; and (iii) the clinical presentations were remarkable, with stupor due to hypercalcemia in case 1, transient ischemic attack in hypertensive crisis in case 2, and appearance of a compressive tumor in the neck for case 3. This case report aims at drawing physicians’ attention to the existence of functional parathyroid cysts, their possible atypical presentations, and discusses the investigation plan and treatment options.

Already in the first description of parathyroid glands in the medical literature, Sandstrom related that “[the structure of the parathyroid gland has] been mentioned as entirely solid […]. This is far from being always the case” [3].

The incidence of parathyroid cysts in the general population is not known precisely. It was very low in a large series of consecutive neck USs (0.075%) [4] and a prevalence of 3% was found in patients operated for cervical mass or hyperparathyroidism [5]. Moreover, a few series have reported on the incidence of functional cysts, which may vary from 10 to 33% [6, 7]. In a cohort of 1700 patients who had undergone surgery for primary hyperparathyroidism, six cases (<1%) of cystic transformation of the parathyroid glands were described, of which five were functional [8]. This large variability in the proportion of functional cysts could be explained by a selection bias from retrospective series and underreporting of this diagnosis [9]. The true incidence of functional and non-functional parathyroid cysts is likely to be higher.

Overall, parathyroid cysts are more commonly encountered between the ages of 40 and 50 years [10, 11] and in women, with a male-to-female sex ratio of 1:2.5. However, male preponderance is observed for functional cysts (male-to-female ratio 1.6:1) [12].

### Origin of parathyroid cysts

Several hypotheses have been brought up to explain the development of parathyroid cysts: (i) vestigial origin and development from the third or the fourth branchial cleft cyst; (ii) coalescence of parathyroid acini; (iii) parathyroid development failure (degenerative cyst) [13] in which remaining parathyroid chief cells start secreting PTH, as seen in case 2 of this report [14, 15]; (iv) retention of secretion vesicles [6]; and finally (v) intra-adenoma hemorrhage with consecutive liquefaction of the hematoma. The three cases reported here are compatible with degenerative cysts, but no other objective parameter can support one hypothesis over the other. Case 2 might be an exception as thymic cells from the third branchial cleft cyst were found to be mixed with lining parathyroid chief cells. Depending on the underlying cause of cyst development and whether they are functional or not, intracystic liquid can be of brownish color or serohemorrhagic [16], and the cyst wall can be lined with thick patches of parathyroid cells [6, 16].

### Clinical presentation

Conditions of discovery of parathyroid cysts are variable. Parathyroid cysts are usually asymptomatic [17] and may be discovered incidentally while performing check-ups for thyroidal or cervical diseases or during neck surgery [18, 19]. Some cysts are discovered during work-up for dyspnea, dysphagia, or hoarseness [20]. Symptomatic hypercalcemia with polyuria, thirst and/or nephrolithiasis can be the first manifestations of a functional parathyroid cyst. Symptoms of overt parathyroid crisis can lead to confusion and even coma [21] and might be due to cyst rupture with discharge of large amounts of PTH into the blood stream. In some cases, signs of hypercalcemia and signs of compression of adjacent structures may coexist [22].

Clinical examination may reveal a palpable mass in the anterior cervical region [18, 23] or laterocervical region [19, 23, 24]. The tumor is usually renitent, smooth, and mobile and can be tightly stuck to the thyroid gland [20]. Therefore, parathyroid cysts can be mistaken for a thyroidal mass [25] or all other cervical tumors.

Non-functional cysts, by definition, do not cause hyperparathyroidism. They contain a clear mucinous fluid [16, 26, 27]; their walls are thin and limited by a flat, cuboid epithelial layer [28, 29].

Parathyroid cysts have been associated with concurrent neck diseases, like parathyroid hyperplasia [16], contralateral parathyroid adenoma with hyperparathyroidism [18, 27], or multinodular goiter in 18% of cases [30], but may just reflect incidental findings.

In more than 85% of cases, parathyroid cysts are found in the neck [29]. They usually present as a single laterocervical deep tumor more commonly of the left side. Multiple cysts have been observed in 3% of patients [16]. The cyst mean size is approximately 3 to 5 cm [14] with a volume of 2 to 75 ml [31], but smaller cysts (< 1 cm) have been described as well. Cervicomediastinal parathyroid cysts can be mistaken for mediastinal goiters [19, 26]. Mediastinal cysts (10 to 15% of cases) up to 12 cm large [26], and functionally active in 42% of cases [18], are mainly found in the anterior mediastinal cavity (82%) and usually originate from inferior parathyroid glands. They are rarely found in the posterior mediastinum, but if they are found in the posterior mediastinum they mostly originate from superior parathyroid glands. In addition, some rare clinical presentations or associations have been described, such as parathyroid cyst in systemic lupus [32] or in type 1 multiple endocrine neoplasia, a condition frequently leading to functional parathyroid adenoma [33].

### Investigations

Cervical ultrasonography is the first choice screening method and usually shows cysts with fine walls. The double-phase parathyroid scintigraphy with ^99m^Tc-sestamibi may be positive in some patients [34] but can be negative even in functional parathyroid gland with high intracystic PTH concentrations, depending on the amount of lining parathyroid tissue. Cases 1 and 3 are two examples of false-negative sestamibi scintigraphy: case 1 being completely negative and case 3 being equivocal with only faint tracer uptake surrounding the cyst. Compressed parathyroid tissue at the cyst’s periphery and/or a lack of significant tracer uptake and retention can explain the false-negative results of scintigraphy [35, 36]. Dilution of the parathyroid tissue content can be another explanation. As ^99m^Tc-sestamibi is used as a nonspecific tumoral tracer, thyroid cysts or nodules may be the cause of such scintigraphic patterns: case 3 illustrates the obvious need to correlate scintigraphy with US, and urges the need for thyroid ^99m^Tc-pertechnetate scintigraphy following the sestamibi late image in patients with equivocal parathyroid scintigraphy. Promising results were found with ^18^F-choline positron emission tomography (PET)-CT in the diagnosis of hyperparathyroidism [37, 38], especially when conventional imaging modalities were inconclusive [39]. It may be especially useful in cases of cystic parathyroid adenoma, but its value is not yet established.

Parathyroid cysts can be mistaken for non-functional thyroid cysts [28, 40]. In that case, fine-needle aspiration and subsequent PTH and thyroid hormones assessment can determine whether the cyst is of parathyroid or thyroid origin, but cannot distinguish between functional and non-functional parathyroid cyst since only plasma PTH and calcium testing can reveal the difference. In a thyroid cyst, intracystic values of thyroid hormones and thyroglobulin are high, in contrast to PTH which is generally undetectable [15, 20, 41]. In case of a parathyroid cyst, PTH levels are usually high [12, 17], reaching several millions pg/ml in some patients (in case 3 – 4,347,000 pg/ml) [1, 17, 20]. The highest intracystic PTH concentration reported in the literature was 7,400,000 pg/ml in a 76-year-old patient [34]. Non-functional parathyroid cyst may also present increased intracystic PTH concentrations, but not as high as in functional cyst. The reason for this difference between non-functional and functional parathyroid cyst is unknown.

In an interesting study comparing 83 solid and 26 cystic parathyroid adenomas, Ghasemi-Rad *et al*. found that patients with solid adenoma had higher calcium and PTH levels and patients with cystic adenoma had higher gland volume and higher phosphate levels [42]. A significant correlation between sestamibi scan, PTH levels, and adenoma volume was observed only in patients with solid adenomas [42].

### Treatment

US-guided fine-needle aspiration can occasionally lead to definitive collapse of the cyst. However, cyst recurrence is regularly observed months after the puncture. Treatment of cystic adenoma by aspiration alone was successful in only 33% of the cases in two series [43, 44]. Sclerosing therapy with intracystic administration of tetracycline or ethanol has been proposed. This technique is, however, penalized by a high risk of uncontrolled induced fibrosis. With only one layer of cells, a parathyroid cyst wall is more prone than thyroid cysts to sclerosing agent leakage in the pericystic tissue. Extension of the fibrotic reaction into the pericystic compartment may cause recurrent nerve damage, mostly when ethanol is used [28]. Of note, failure of sclerosing therapy would increase risks and morbidity of any subsequent surgical procedure.

Indication and timing of surgical procedure in connection with sclerosing therapy have been largely debated. In a limited series of 14 patients, Clark proposed that surgery should be performed only after failure of fine-needle puncture or for mediastinal and retromandibular cysts [12]. In four patients (29%), surgery was necessary because of cyst recurrence 6 to 48 months after the initial puncture. Surgery for a cystic parathyroid gland as first-line treatment appears to be the best therapeutic approach for several reasons [12]: (i) although rare, the possibility of a cystic parathyroid carcinoma should not be missed because of the dramatic consequences of an inappropriate procedure [45]; (ii) uncontrolled extension of pericystic fibrosis may entrap and harm the recurrent nerve and in addition may compromise or at least complicate further surgery [46]; (iii) in the hands of a skilled endocrine surgeon, parathyroidectomy is rapid, safe, and efficient. Lastly, exploration of the four parathyroid glands may be indicated if intraoperative plasma PTH levels do not drop appropriately after removal of the cystic parathyroid gland [47].

### Algorithm for parathyroid cysts

**Fig. 4:**
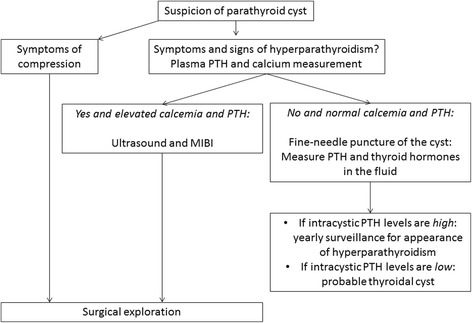
Proposed investigation and treatment algorithm in case of suspected parathyroid cyst. *MIBI* ^99m^Tc sestamibi, *PTH* parathyroid hormone

We propose the following investigations and treatment plan when a cystic parathyroid gland is suspected (Fig. 4). First, the function of the cyst should be determined by the patient’s history and by measurement of plasma PTH and calcium levels (ionized calcium if available). If the cyst is functional, US of the neck and double-phase parathyroid scintigraphy with SPECT-CT and thyroid scintigraphy should be obtained in order to possibly confirm the location of the cystic gland. At this stage, we do not recommend a cyst puncture, even as a diagnostic tool. The cystic gland should be surgically removed and perioperative assessment of PTH levels in plasma should be obtained. If plasma PTH levels drop, the patient is cured. If plasma PTH does not decrease, exploration of the remaining glands should be performed during the same procedure. Macroscopic examination can already be suggestive (Figs. 1b and 3c) but frozen sections will definitively confirm the diagnosis.

Of note, the presence and responsiveness of calcium-sensing receptors to cinacalcet is unknown and we found no report on the use of cinacalcet in treating functional cystic parathyroid gland.

If the cyst is non-functional, it should be evaluated to distinguish it from a thyroid cyst. Fine-needle puncture and analysis of the fluid for thyroid hormones should be performed, including PTH dosage. If intracystic PTH levels are high and the patient has no sign of hyperparathyroidism, including normal plasma and calcium levels, yearly surveillance is advisable. If PTH is undetectable, patients should be considered to have thyroidal cyst and followed up accordingly. If symptoms are caused by the size or position of the cyst (tumor in the neck, hoarseness, pain, and so on), it should be surgically removed. So, patients with cystic lesion of the neck should be asked for symptoms and signs of hyperparathyroidism, with the knowledge that the disease has an extremely large and variable presentation.

We must admit, however, that this proposal for investigations and treatment of cystic adenomas has not been validated and urge a large multicentric prospective study.

## Conclusions

Parathyroid cyst should be evoked, and signs and symptoms of hyperparathyroidism actively searched for when facing any cystic tumor in the anterior neck or the upper mediastinum. The best investigation for a functional cyst defined by hyperparathyroidism is US and sestamibi scan in order to localize the functional gland before surgery. US-guided fine-needle puncture should not be recommended in this setting. In cases of non-functional cyst, US-guided fine-needle puncture with analysis of both thyroid hormones and PTH levels will determine the further management of the cyst.

## Abbreviations

25-OH vitamin D: 25-Hydroxyvitamin D
^99m^Tc: Technetium 99m
BP: Blood pressure
CT: Computed tomography
PTH: Parathyroid hormone
Sestamibi: Sesta methoxyisobutylisonitrile
SPECT: Single-photon emission computed tomography
US: Ultrasound
